# Case report: Treatment of parkinsonism secondary to ciltacabtagene autoleucel using a combination dopaminergic regimen

**DOI:** 10.3389/fimmu.2024.1444010

**Published:** 2024-09-20

**Authors:** Raya Aliakbar, Olga Manouvakhova, Cindy Wong, Myo Htut, Johannes Pulst-Korenberg, Murali Janakiram, Michael Rosenzweig, Scott R. Goldsmith, Xenos L. Mason

**Affiliations:** ^1^ Department of Neurology, University of Southern California Keck School of Medicine, Los Angeles, CA, United States; ^2^ Division of Multiple Myeloma, Department of Hematopoietic Cell Transplantation, City of Hope Comprehensive Cancer Center, Duarte, CA, United States

**Keywords:** ciltacabtagene autoleucel, movement and neurocognitive toxicity, parkinsonism, CAR-T, multiple myeloma

## Abstract

We report on a patient with ciltacabtagene autoleucel-induced movement and neurocognitive toxicity, which was refractory to immunosuppression but responsive to combination dopaminergic therapy (carbidopa/levodopa, ropinirole, amantadine). Response was seen upon both initial treatment and rechallenge after unintended withdrawal. This is the first report of a successful symptomatic treatment of this well-described neurotoxic syndrome.

## Introduction

Neurotoxicities have been described with B-cell maturation antigen (BCMA)—targeting chimeric antigen receptor (CAR) T-cell therapy for multiple myeloma (MM). The most challenging is a subacute parkinsonism termed “movement and neurocognitive toxicity” (MNT) most frequently associated with ciltacabtagene autoleucel (cilta-cel) (1). In CARTITUDE-1, the reported incidence of MNT was 5.2%, with limited or no improvement following immunosuppression or symptomatic management with carbidopa/levodopa (2). Graham and colleagues recently reported successful management of MNT with high-dose cyclophosphamide (2 g/m^2^), although attempts at symptomatic management were not reported (3). Although effective, such aggressive immunosuppression predisposes to opportunistic infections. Herein, we report on a patient with MNT who did not respond to immunosuppression but did respond to a combination of dopaminergic regimen (carbidopa/levodopa, ropinirole, amantadine) with initial treatment and upon rechallenge after unintended withdrawal.

## Case description and diagnostic assessment

A 67-year-old man with Revised International Staging System stage II MM with 1q gain and predominantly oligo-secretory extramedullary disease presented after receiving cilta-cel as sixth-line therapy. He was initially diagnosed 3.5 years prior and had an aggressive extramedullary relapse 6 months after autologous stem cell transplantation. His past medical history was notable for glaucoma, benign prostatic hypertrophy, and chemotherapy-induced sensory neuropathy, and his medications at the time of evaluation included acyclovir, aspirin, brimonidine, famotidine, finasteride, gabapentin, transdermal lidocaine, latanoprost, morphine IR, and senna. There was no family history of hematologic malignancy, Parkinson’s disease, tremor, or other neurodegenerative disorders.

He achieved transient partial responses to DT-PACE, carfilzomib–pomalidomide–dexamethasone, and daratumumab but developed retro-orbital and progressive perinephric plasmacytomas and so was referred for CAR T consideration. Despite peripheral lymphocytopenia at leukapheresis (absolute lymphocyte count [ALC] <100 cells/μL), production of the cilta-cel product at a commercial dose of 0.6 × 10^6^ CAR+ T cells was successful. He required bridging radiotherapy, along with bortezomib–dexamethasone and selinexor, but progressed through bridging.

He proceeded with standard fludarabine–cyclophosphamide lymphodepletion followed by cilta-cel infusion on day (D) 0. On D+7, he developed grade 2 cytokine release syndrome (CRS) and cognitive impairment (defined as grade 2 immune effector cell-associated neurotoxicity syndrome (ICANS)) for which he received one dose of tocilizumab and 3 days of dexamethasone with resolution by D+10. Notably, between D+9 and D+11, his ALC increased from 60 cells/μL to 1,340 cells/μL. He was discharged on D+14.

**Figure 1:**
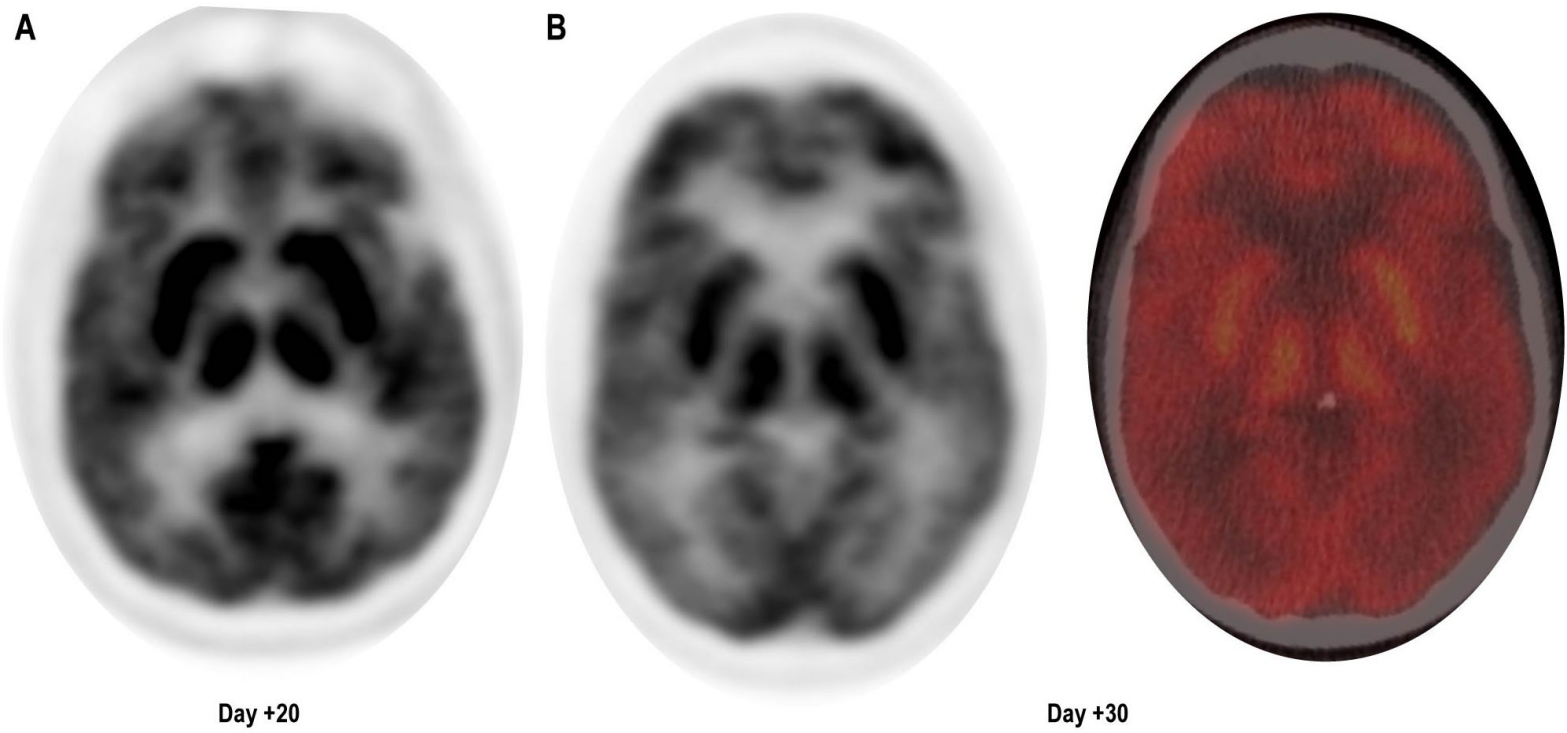
Brain (18) F-fluorodeoxyglucose (FDG) positron emission tomography (PET) CT demonstrating diffuse symmetrically enhanced tracer uptake in the caudate, putamen, and thalamus. **(A)** Day + 20, unfused PET. **(B)** Day +30, unfused PET (left) and fused color image (right).

At follow-up, on D+19, he was readmitted with 1 day of confusion, slowness, and falls, concerning for MNT. Examination results demonstrated apathy, masked facies, bradykinesia, and upper-extremity resting tremor. ALC was 2,030 cells/μL and cerebrospinal fluid (CSF), demonstrating 71 white cells/μL all CD3^+^ by flow cytometry. Infectious and inflammatory encephalitis workup in blood and CSF was unremarkable. CT of the head was found to be unremarkable for intracranial abnormalities, and due to the presence of stapes prostheses, a brain MRI was not performed. Brain (18) F-fluorodeoxyglucose (FDG) positron emission tomography (PET) CT demonstrated diffuse and symmetrically increased FDG activity in the bilateral caudate, putamen, and thalami (max SUV 10.91 relative to cortical activity, **Figure 1**). Concurrent whole-body FDG PET, serum biomarkers, and bone marrow biopsy demonstrated a stringent complete response. There being no evidence of delirium, significant metabolic derangement, or infection, his diagnosis was felt to be consistent with MNT. The acute time course was inconsistent with idiopathic Parkinson disease or other neurodegenerative parkinsonism, and no dopamine antagonists had been administered to suggest the possibility of drug-induced parkinsonism.

After administration of intravenous immunoglobulin G (2 g/kg) and 10 mg of dexamethasone every 8 h, severe apathy persisted. Carbidopa/levodopa 25 mg–100 mg was initiated at one tablet three times a day, which resulted in some early improvement in rigidity and bradykinesia but little improvement in apathy. On D+27, intravenous cyclophosphamide at 2 g/m^2^ was administered as per Graham et al. (3). Despite a rapid decrease in ALC, his MNT-related apathy worsened, manifesting as disinterest in medical care, nutrition, and hygiene, and decreased communication with family and caretakers. Repeat CSF was acellular, and the CSF autoimmune encephalitis panel and CSF meningitis/encephalitis BioFire and PCR pathogen panel were negative. Repeat brain FDG PET/CT demonstrated increased FDG uptake in deep gray nuclei, stable from prior. On D+61, he was transferred to a tertiary neurology center with movement disorder specialization for further evaluation.

On transfer, his examination demonstrated mild midline and appendicular rigidity, and moderate generalized and appendicular bradykinesia manifested by slowness and decrement of repeated movements. Detailed testing was limited by his apathy and inattention; he would participate for less than 5 s–10 s before withdrawing by closing his eyes and giving short or diminished responses. Recent and remote memory, language, and visuospatial construction were intact without the presence of apraxia, agraphesthesia, or any agnosia.

Carbidopa/levodopa 25 mg/100 mg was uptitrated to three tablets three times daily with improvement in rigidity, but persistent bradykinesia and apathy. The dopamine agonist ropinirole was then initiated with titration from 1 mg to 2 mg three times daily with notable improvement of bradykinesia and apathy and resolution of rigidity. Daytime somnolence was successfully treated with amantadine 100 mg twice daily. This combination regimen resulted in improvement and near-resolution of his MNT symptoms, facilitating discharge to a skilled nursing facility.

The efficacy of this regimen was confirmed through involuntary withdrawal and rechallenge as follows: the patient remained pancytopenic and immunosuppressed and developed acute hypoxia and pneumonia on D+82. Subsequent bronchoalveolar lavage demonstrated respiratory syncytial virus, methicillin-resistant Staphylococcus aureus, and mucor. His oxygen requirements (bilevel positive airway pressure, BiPAP), hypoxemic encephalopathy, and respiratory distress limited enteral access, thereby preventing the administration of his dopaminergic medications, leading to the recurrence of MNT symptoms. Reliable enteral access was regained through a percutaneous gastrostomy tube, and his hypoxemia and encephalopathy improved. His prior dopaminergic regimen was then readministered, which once again improved his apathy, bradykinesia, and rigidity. Despite neurologic improvement, he developed sepsis and massive hemoptysis on D+94 and expired shortly thereafter. For summary of the clinical course, see **Table 1**.

**Table 1:** Summary of clinical time-course.

CAR-T day	+7	+8 to +10	+19	+27	+28	+29	+30 to +60	+61 to +82	+83 to +91	+92 to +107	+108
**ALC**	40	20–43	2,030	1,030	1,400	920	40–500	100–170			
**Events**	Gr. II CRS; Gr. II ICANS		Re-admit						Pneumonia Resp. failure	Regained enteral access (PEG)	Hemoptysis, Sepsis, Death
**MNT status**											
**Immunotherapies**	Toci.		IVIg								
**Steroids**	Dex.	Dex.	Dex.	Dex.	Dex.	Dex.	Dex.	Dex.			
**Dopaminergic therapies**					Carbidopa/levodopa (C/L)	C/L	C/L	C/L Ropinirole Amantadine		C/L Ropinirole Amantadine	C/L Ropinirole Amantadine

ALC , Absolute Lymphocyte Count; Dex., Dexamethasone; PEG , Percutaneous Endoscopic Gastrostomy; Toci. , Tocilizumab. Shading represents MNT symptoms, where darker shading represents more severe symptoms.

## Discussion

There is a paucity of guidance on management of MNT. The association of these symptoms with higher-grade CRS, ICANS, and robust CAR T expansion suggests that acute basal ganglia injury may precede later development of MNT (4). Indeed, pre-CAR T cytoreduction and early mitigation of CRS and ICANS have reduced the incidence of MNT (1). Once symptoms occur, however, aggressive immunosuppression/lymphocyte depletion in blood/CSF may not be successful and carry a risk of severe infections and cytopenias. This case demonstrates the potential for symptomatic management with a combination dopaminergic regimen with integrated involvement of neurologic movement disorder specialists. The risk profiles of immunosuppressive versus symptomatic dopaminergic strategies may favor a trial of symptomatic management in conjunction with first-line immunosuppressive treatment, although further prospective study is needed. Should a clinical improvement be noted, based on the authors’ experience with idiopathic Parkinson, we suggest continuation of this regimen for 3 months post peak CART-cell expansion, or symptom onset (whichever is later), followed by a challenge of down-titration over 7–14 days. Longer-term follow-up, precluded by this patient’s death, should be noted and reported in the future.

Oeklen et al. demonstrated BCMA expression on neurons and astrocytes in the basal ganglia of a postmortem MNT patient, suggesting the on-target, off-tumor effect of infiltrating CAR T cells (4). These studies have yet to implicate the site of damage along the dopamine transport axis. Like other reports, carbidopa/levodopa monotherapy was ineffective in our case (1). However, our patient did respond to dopaminergic agonists, suggesting that CAR T lymphocytic basal ganglia infiltrate may preferentially target the presynaptic terminals of dopaminergic synapses. Upon administration, levodopa crosses the blood–brain barrier, where it is taken up into dopaminergic neurons by the dopamine transporter, converted to dopamine by the enzyme L-aromatic amino acid dopa-2decarboxylase, and packaged by the vesicular–monoamine–transporter (5). After synaptic release, it is either metabolized or undergoes reuptake into the presynaptic dopaminergic terminal. We hypothesize that in CAR T-mediated MNT, inflammation directed against the presynaptic terminal may lead to dysfunction in any or all of these processes (i.e., through directed immunotoxicity toward the dopamine transporter). Ropinirole acts by stimulating post-synaptic D2-like dopamine receptors, thereby bypassing any presynaptic dysfunction (6). In this case, non-motor MNT symptoms were responsive to dopaminergic therapy, a distinguishing feature from Parkinson’s disease, which often requires adjunctive serotonergic and cholinergic therapies for treatment of these symptoms. The few cases of MNT that have included examination by a Dopamine Transporter Single-Photon-Emission-CT (DaT SPECT) have reported normal presynaptic dopaminergic terminal density (1, 4, 7); however, interpretation of these scans can be challenging in the absence of asymmetric dopaminergic degeneration (as is usually seen in idiopathic PD). Moreover, functional properties of presynaptic dopaminergic terminals are not assessed by DaT SPECT. Previous MNT case reports and case series have reported no benefit from carbidopa/levodopa, although none have detailed trials of dopamine agonists or amantadine (1, 4, 7).

This case highlights the potential efficacy of combination dopaminergic therapy in MNT, even in cases that fail to respond to immunosuppression. Although long-term follow-up evaluation and testing was not possible, clinical observations following medication withdrawal and readministration support a causal role for this regimen in the observed improvement in MNT symptoms. We have suggested multiple hypotheses, which may contribute to future study of the immunopathogenesis and treatment of this condition.

## Data Availability

The original contributions presented in the study are included in the article/supplementary material. Further inquiries can be directed to the corresponding authors.
